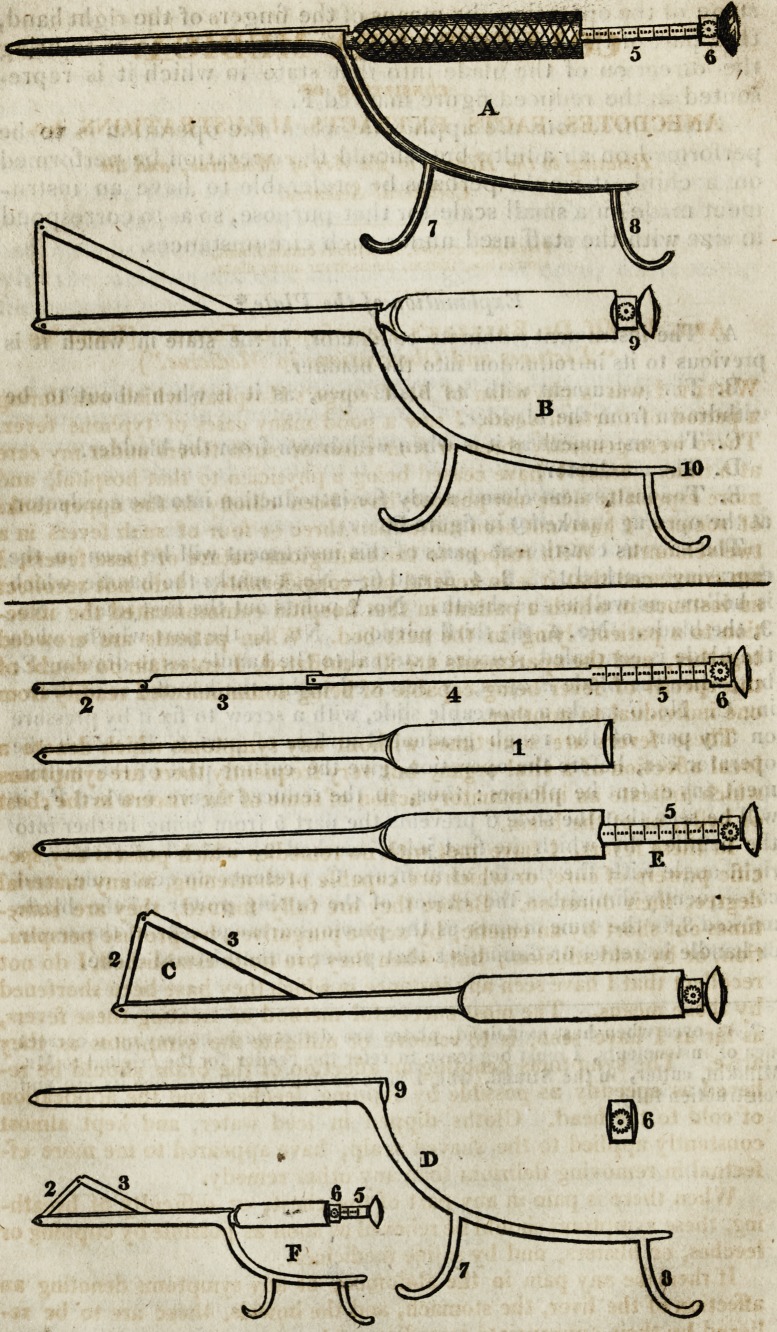# Description of an Instrument, Proposed as an Improvement on the Lithotome Caché of Frère Cosme

**Published:** 1826-01

**Authors:** Andrew Blake

**Affiliations:** Member of the Royal College of Surgeons, and Surgeon to his Majesty's 7th Dragoon Guards.


ART. VIII.—*Description of an Instrument, proposed as an Improvement on the Lithotome Caché of* Frère COSME. By ANDREW BLAKE, M.D. Member of the Royal College of Surgeons, and Surgeon to his Majesty's 7th Dragoon Guards.

[With an Engraving.]

IN proposing a new, or rather an improvement on an old instrument, for the performance of the operation for the stone, I do not consider it necessary to enter into a particular description of the different modes hitherto devised for attaining the same end: neither does it appear to me that I am called upon to detail the comparative advantages of the various instruments invented at different epochs for that purpose. It will, I trust, be deemed sufficient to state, that some of the most eminent surgeons, both in their lectures and practice, give the preference to the scalpel for the performance of the lateral operation. Unfortunately, however, we are not all blessed with the dexterity of a COOPER, a GUTHRIE, or the late much-lamented Mr. DEASE, of Dublin; and, in consequence, the use of the scalpel is still confined to a very limited number of operators. My present object, therefore, is to offer to the profession an instrument possessing, in a high degree, the advantages of the scalpel, and at the same time free from the dangers and difficulties attending the use of the bistouri, or lithotome caché, invented by Frère COSME; an instrument deservedly regarded, by all continental as well as by British surgeons, as perhaps the least objectionable, and best calculated for general use, of any hitherto contrived. To it, however, it is well known many serious objections have been made. First, it is evident that its motion, while opening, or rather while the blade is diverging from its sheath, is more of a compressive, and consequently of a tearing, than of a cutting nature: hence it is more liable to be followed by inflammation, and less likely to divide the prostate sufficiently; that gland being calculated, from its structure, to recede before the edge of the instrument. Secondly, its point, if not rounded considerably, (in which case it may not cut its way out,) may wound the bladder at its fundus or sides, and thereby give rise to infiltration of urine into the neighbouring parts, and thus occasion inflammation, and perhaps gangrene. And lastly, from the manner in which the instrument is generally used,—namely, without a proper director,—considerable knowledge, care, and dexterity are required, on the part of the operator, to avoid wounding the rectum, or internal pudic artery.

When we consider that the bistouri, or lithotome caché, is a French invention, and at the same time read the following paragraph from the pen of a French author, we may feel convinced of the truth of the dangers depicted as inseparable from its use: —"Il est vrai qu'on peut léser le rectum et les vaisseaux honteux, si l'incision est faite trop en dedans, ou trop en dehors; que le bas-fond de la vessie peut être ouvert, si l'on élève trop le poignet, et les parois de ce viscère percées d'outre en outre, si l'on enfonce l'instrument d'une manière trop brusque, et avec trop de force."—Richerand.

A slight examination will, I hope, convince most surgeons that the dangers above stated as attendant on the use of the bistouri caché, have been obviated in the construction of the instrument of which a plate is subjoined.

It will be observed, in the instrument I propose,—First, that the progressive motion forward and outward of the blade, while it is assuming the position intended to be given it for the performance of the operation, enables it to divide, without the least violence, all that part of the prostate gland and neck of the bladder that may present itself to its edge. Secondly, that, by the mode in which the instrument opens, it tends, with its first joint, (marked No. 2 in the figures C and F,) to push the coats of the bladder before it, should they come in contact with it, and thereby prevents the possibility of their being wounded by the blade. And lastly, the director, which, during its introduction into the bladder, forms an integral part of the instrument, becomes a safe and steady guide for the opening and withdrawing of its cutting portion, by its enabling the operator to hold the whole, with the left hand, in a proper direction, and close to the arch of the pubis; thereby preventing any probable risk of wounding either the rectum or internal pudic artery, while the division of the prostate gland and neck of the bladder is completed, with comparatively little violence.

I shall here submit the only objection which has been made to me concerning the facility of using this instrument,—viz. that, should it be intended to make an opening, let us suppose, of an inch and a half in the neck of the bladder, the point of the instrument must be introduced to that extent at least beyond that part, previously to our being able to open it. However, I should hope, when it is considered that the ordinary diameter of the bladder far exceeds the extent of any opening ever necessary for the extraction of a stone, such an objection will be found merely theoretical. Perhaps this circumstance, on the contrary, ought to be regarded rather as an advantage, as it ensures the necessity of a sufficient introduction of the instrument into the bladder previously to its opening; it having happened more than once that even an experienced operator, when using the scalpel, fancied he had got into the bladder, when he had merely cut the parts exterior to it: and there is no reason why the same unpleasant mistake might not occur while using the bistouri caché.

### *Directions for using the Instrument.*

In performing the lateral operation with this instrument, we are to suppose the previous steps of the operation completed, as if we were about to use the common bistouri caché, or the cutting gorget; we then take hold of it, as it is represented in figure A, in the right hand, and introduce it into the bladder as if it were the bistouri caché; and having ascertained, by the flow of urine, and by its touching the stone, that it is fairly in that viscus, we are to withdraw the staff, and place the fore and middle fingers of the left hand on the part of the director marked 7, and the thumb on that part of it marked 8, by which we obtain a firm hold of it. Having done so, we raise the left hand, by turning the little finger towards the right groin of the patient, so as to give the cutting part of the instrument the exact direction we wish; while, with the right hand, we push in the graduated handle marked 5 and 6, as far as it will go. At this period the instrument is open in the bladder, (having divided all that part of the prostate gland and neck of the bladder which opposed it,) and in that state in which it is represented in figure B; so that it only remains for us to continue to hold the director steadily, with the left hand, close against the arch of the pubis, and to give the blade of the instrument the proper obliquity, so as to avoid the rectum and internal pudic artery; while we withdraw, with the right hand, the instrument, as it is represented in figure C. When we are about to withdraw the lithotome from the director, if we place the top of the right thumb, and press for a moment against the point of the director marked 10, in figure B, we shall find that we facilitate considerably the disengagement of the instrument.

Should the operator wish, after having cut through the neck of the bladder and prostate gland, to diminish the extent of the cutting power of this instrument, on the principle recommended by BOYER in France, and Mr. GUTHRIE in this country, in order to avoid, with more certainty, the risk of wounding the pudic artery, he can very easily do so by withdrawing, at this stage of the operation, by means of the fingers of the right hand, that part of the instrument marked 6 in figure B, so as to bring the direction of the blade into that state in which it is represented in the reduced figure marked F.

These directions are applicable when the operation is to be performed on an adult; but, should the operation be performed on a child, it would perhaps be preferable to have an instrument made on a small scale for that purpose, so as to correspond in size with the staff used under such circumstances.

### *Explanation of the Plate.**

A. The instrument within its conductor, in the state in which it is previous to its introduction into the bladder.

B. The instrument with its blade open, as it is when about to be withdrawn from the bladder.

C. The instrument as it is when withdrawn from the bladder.

D. The conductor.

E. The instrument closed ready for introduction into the conductor, at the opening marked 9 in figure D.

The various constituent parts of this instrument will be seen in the engraving, marked 1, 2, 3, 4, 5, and 6.—No. 1 marks the handle, which is hollow, as well as the sheath. No. 2 points out the first joint. No. 3, the blade. No. 4, the third portion. No. 5, the part which, when the blade is concealed, remains external to the handle, as in drawing E; but, when the blade is open, is received within the handle, as in drawing C. No. 6 marks a moveable slide, with a screw to fix it by pressure on any part of the rough graduated surface of part 5, by which the operator can, before the operation, give the cutting part of the instrument any extent he pleases: thus, in the reduced figure marked F, it will be seen that the slide 6 prevents the part 5 from going further into the handle; by which the first joint, marked 2, instead of forming a right angle with the sheath, as in figure C, presents an acute one, and consequently diminishes the extent of the cutting power of the blade marked 3, in the same manner as the previous arrangement of the screw or handle increases or diminishes that power in the bistouri caché.

---

* As, even when best explained, plates are defective in conveying a correct idea of instruments, I must beg leave to refer the reader for the original to Mr. Millikin, cutler, in the Strand, who, I have no doubt, will describe its use and construction fully.

## Figures and Tables

**A B E C D F f1:**